# Supplementary material for: Selection of start codon during mRNA scanning in eukaryotic translation initiation
Source: Commun Biol. 2022 Jun 15;5:587. doi: 10.1038/s42003-022-03534-2 (PMC9200866; doi:10.1038/s42003-022-03534-2)
Supplement: Supplementary file 2 — Supplementary Information [file 42003_2022_3534_MOESM2_ESM.pdf]

Supplementary Information for

Selection of start codon during mRNA scanning in eukaryotic translation initiation

Ipsita Basu[1], Biswajit Gorai[1,†,£], Thyageshwar Chandran[2,†,#], Prabal K. Maiti[1,*] and Tanweer Hussain[2, *]

[1]Center for Condensed Matter Theory, Department of Physics, Indian Institute of Science, Bangalore-560012, India.
[2]Department of Molecular Reproduction, Development and Genetics, Division of Biological Sciences, Indian Institute of Science, Bangalore-560012, India.

† Both authors contributed equally to this manuscript

[#]Present Address: Department of Biotechnology, National Institute of Technology-Warangal, Telangana-506004, India.

[£]Present address: Department of Chemical Engineering, University of New Hampshire, Durham, NH-03824, USA.

*Corresponding author e-mail: hussain@iisc.ac.in, maiti@iisc.ac.in

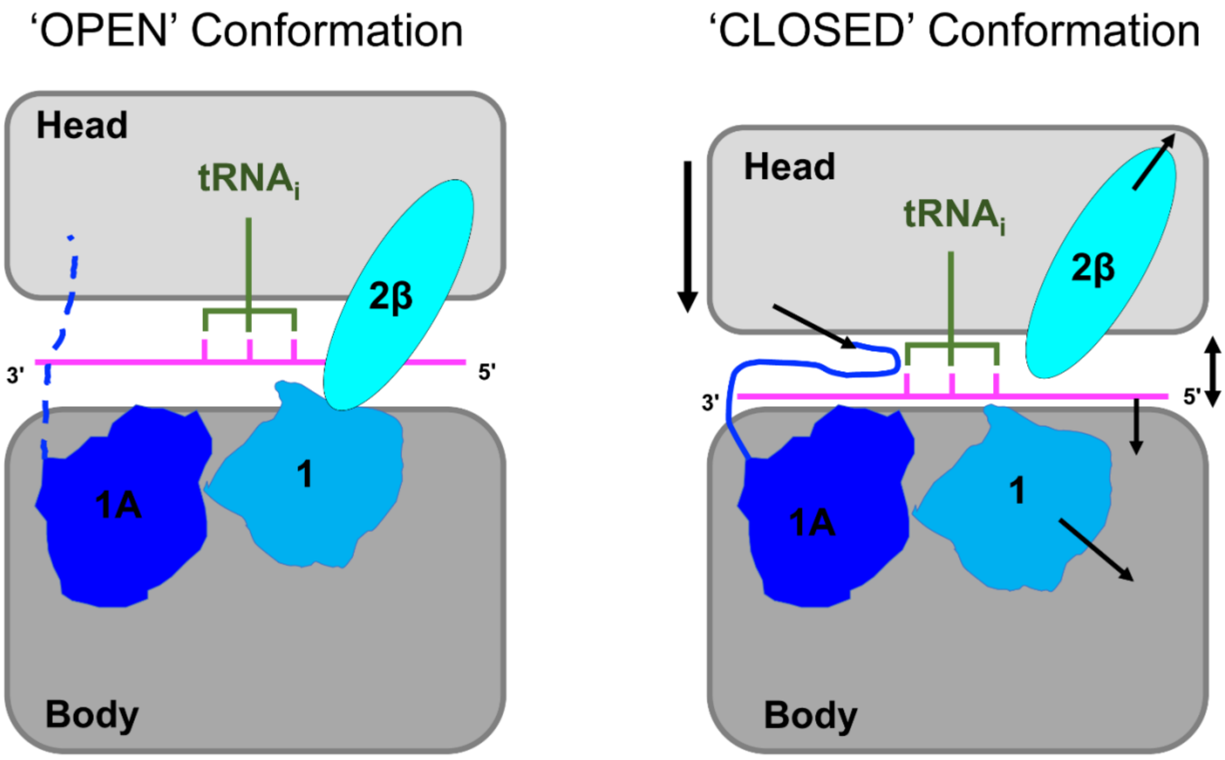

Supplementary Figure 1: Schematic of open and closed conformation of 48S PICs highlighting the major differences between the two. The arrows shown in the closed conformation points out the differences in conformation, namely,

1. the 40S head moves down closer with respect to the body with attendant compression of rRNA helix 28,
2. the mRNA channel and the P site are narrowed,
3. the mRNA and tRNAi positioned in the P site ~7 Å closer to the 40S body compared to that found in py48S-open complex,
4. eIF1 undergoes subtle repositioning away from original binding position on transition to the closed state to accommodate $tRNA_i$ in the PIN conformation,
5. the N-terminal tail (NTT) of eIF1A is observed in proximity to the codon:anticodon duplex, and
6. eIF2β moves away from eIF1 in closed conformation.

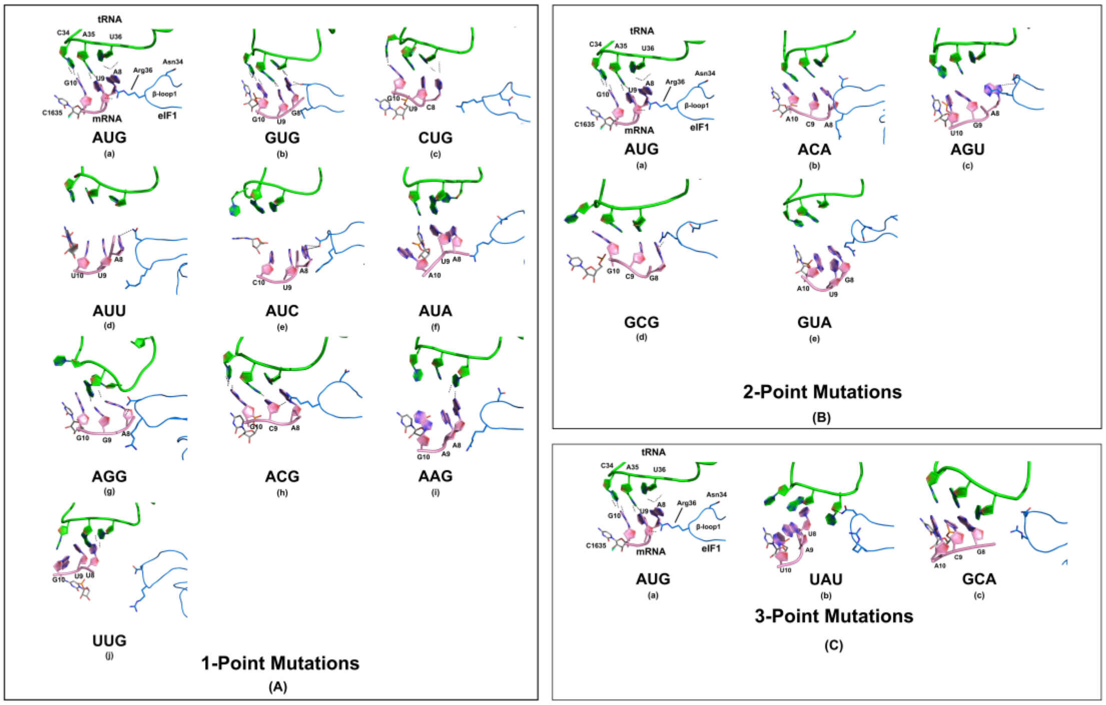

Supplementary Figure 2: Cartoon representation of codon-anticodon interactions of MD average structures (last 40 ns) of AUG, (*A*) 1-point mutations, (*B*) 2-point mutations and (*C*) 3-point mutations. $tRNA_i$ is shown in green, mRNA in pink, eIF1 in blue and C1635 of rRNA, which provides the stacking interaction to codon-anticodon is shown in grey colour. Arg36 and Asn34 of eIF1 are shown in stick representations. AUG is shown as reference in all the three panels.

In panel A, clear base pairing could only be observed for GUG, CUG, UUG and ACG. For panel B (2-point mutations) and C (3-point mutations) no base pairing could be observed.

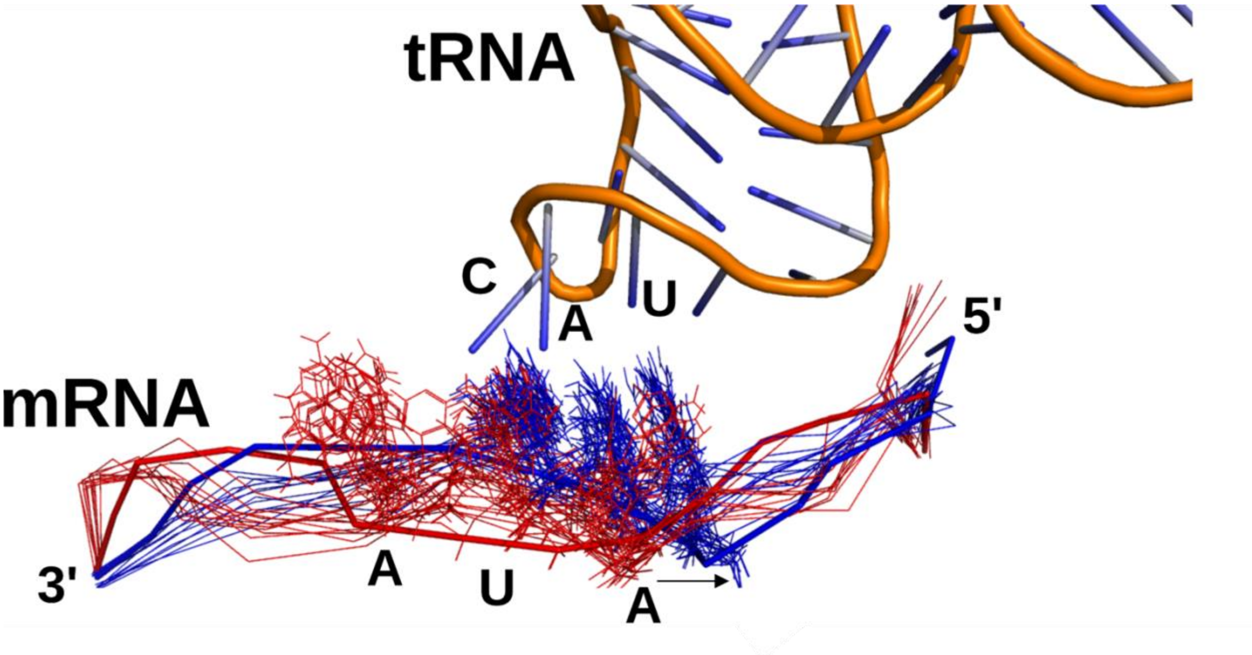

Supplementary Figure 3: Base-pairing of codon:anticodon in AUA simulation runs.
The figure represents sequence of snapshots of mRNA extracted from the AUA simulation runs with (red) and without (blue) eIF2α. The codons of AUA are shown to indicate a shift in position towards the E site (depicted by an arrow) in the absence of eIF2α (blue) simulation run. The $tRNA_i$ is shown in cartoon representation to indicate the P site.

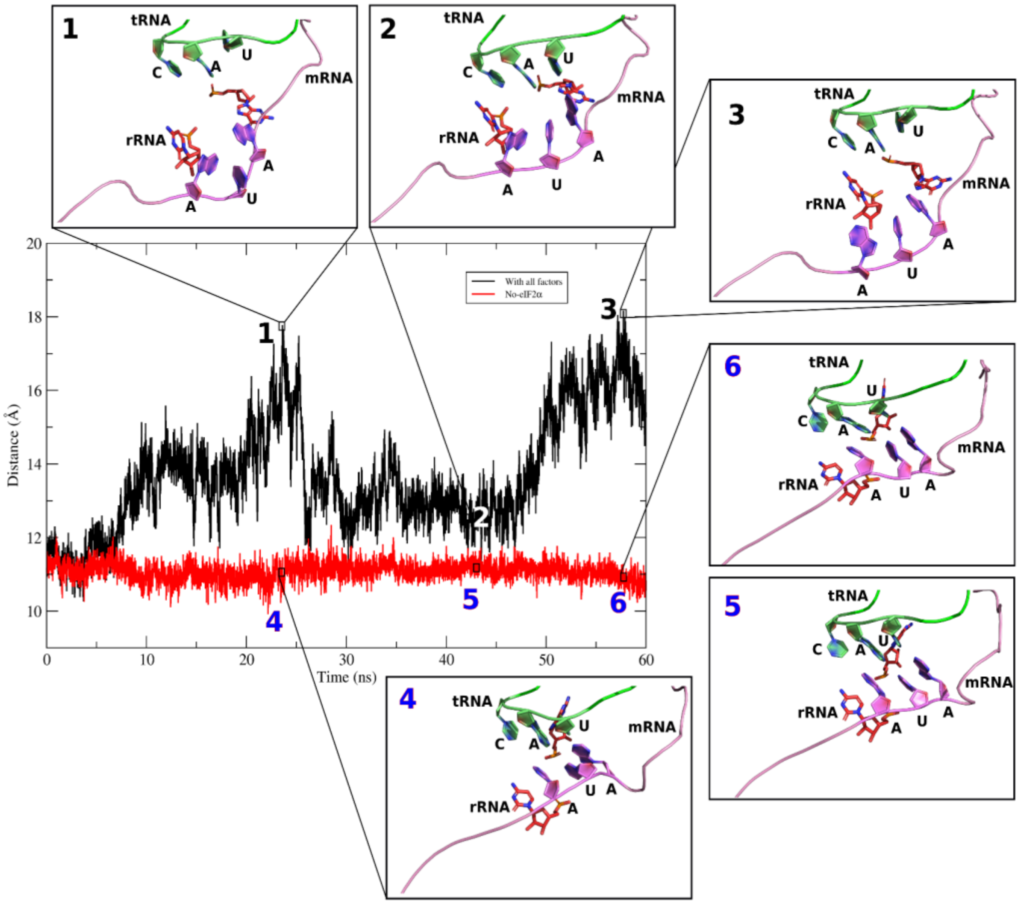

Supplementary Figure 4: **Base-pairing of codon:anticodon in AUA run in the absence of eIF2α.**

The plot represents distance between the centre of mass of codon and the centre of mass of anticodon nucleotides of the AUA MD simulation trajectory in the presence (black lines) and in the absence of eIF2α (red lines). Panel 1-3 represents the snapshots (with larger observed fluctuations) extracted at 1) t=24 ns; 2) t=43 ns ; 3) t=58 ns of AUA MD simulation trajectory in the presence of eIF2α and panel 4-6 represents the snapshots extracted at the same time line from MD simulation run of AUA in the absence of eIF2α.

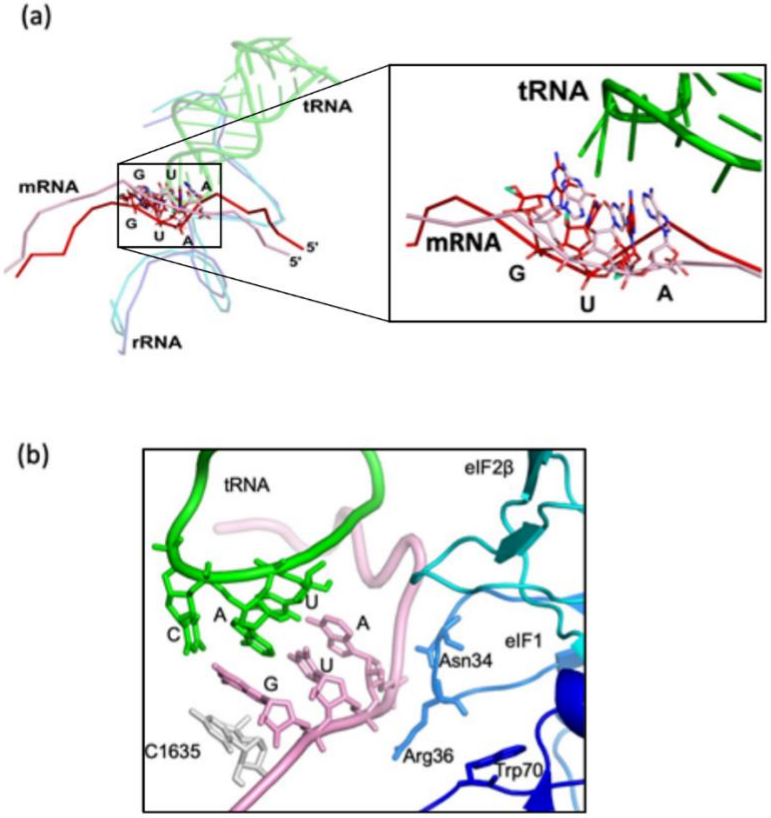

Supplementary Figure 5: Recognition of AUG start codon during scanning

(a) The mRNA (and start codon) in average MD structure of AUG simulation run in open conformation (red) occupies a different position when compared with mRNA in 48S PIC in closed state (PDB ID: 6FYX (pink). The PDBs were superposed onto each other based on the rRNA (1147-1174; chain 2) from the body of the 40S ribosome. The rRNA from AUG simulation run is shown in dark blue whereas rRNA from PDB ID: 6FYX is in light blue. The $tRNA_i$ (green) at the P site is from average MD structure of AUG simulation run in open conformation. An enlarged view of the codon:anticodon is on the left.

(b) Asn34 of eIF1 interacts with the first nucleotide A of the codon in another average MD structure with AUG codon.

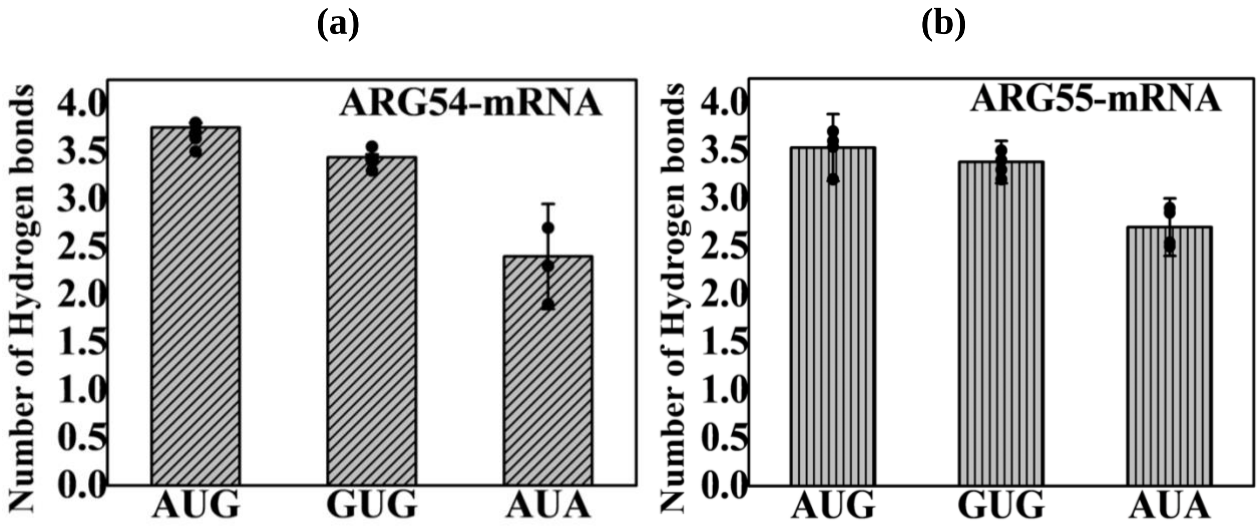

Supplementary Figure 6: Average number of hydrogen bonds between the Arg residues of eIF2α and the mRNA nucleotides, calculated for the a) Arg54 and b) Arg55 for AUG, GUG and AUA simulation runs. Error bar arises due to averaging over the independent runs of each system. Points shown on the plots were the data used to make the histogram.

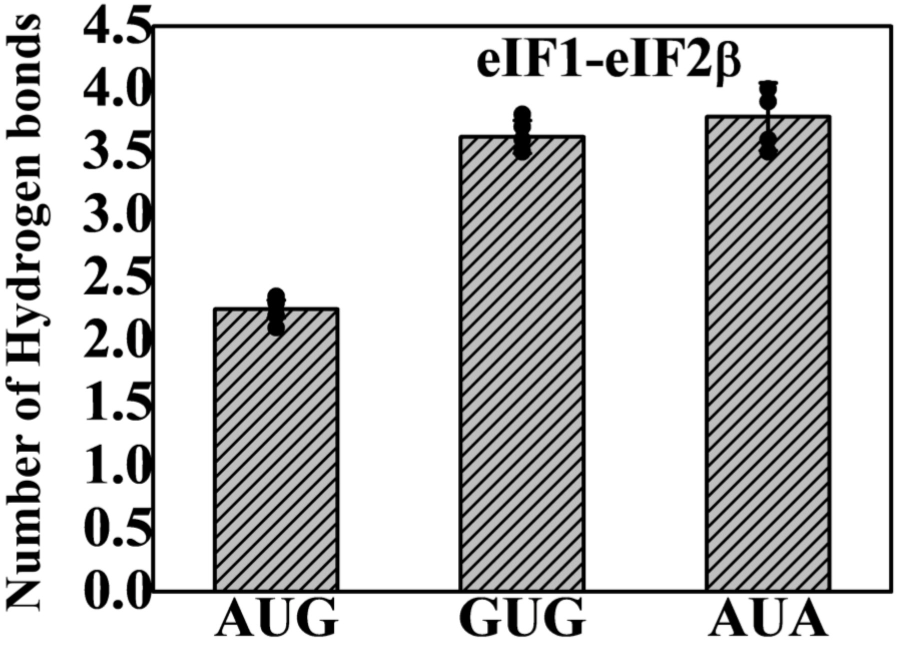

Supplementary Figure 7: Average number of hydrogen bonds with error bar (averaged over the four set of independent runs) between eIF1 and eIF2β interface for AUG, GUG and AUA runs. The decrease in the number of hydrogen bonds for cognate codon (AUG) is noticed compared to GUG and AUA codons. Points shown on the plots were the data used to make the histogram.

Supplementary Table 1: Binding energy values (kcal/mol) for different codons:anticodons

CODON	Binding energy (kcal/mol)	Relative binding energy (kcal/mol)	Average
**AUG**	RUN1: -22.24<br>RUN2: -19.7<br>RUN3: -23.72<br>RUN4: -20.03		-21.42
**GUG**	RUN1: -20.8<br>RUN2: -21.19<br>RUN3: -19.68<br>RUN4: -19.22	0.62<br>0.23<br>1.74<br>2.2	1.19
**UUG**	RUN1: -19.13<br>RUN2: -20.88<br>RUN3: -17.68<br>RUN4: -16.8	2.29<br>0.54<br>3.74<br>4.62	2.79
**CUG**	RUN1: -21.09<br>RUN2: -19.85<br>RUN3: -17.48<br>RUN4: -15.65	0.33<br>1.57<br>3.94<br>5.77	2.9
**ACG**	RUN1: -19.7<br>RUN2: -16.63<br>RUN3: -18.00<br>RUN4: -14.77	1.72<br>4.79<br>3.42<br>6.65	4.14
**AGG**	RUN1: -9.89<br>RUN2: -5.00<br>RUN3: -10.30<br>RUN4: -11.61	11.53<br>16.43<br>11.12<br>9.81	12.22
**AAG**	RUN1: -6.30<br>RUN2: -7.41<br>RUN3: -10.30<br>RUN4: -5.61	15.12<br>14.01<br>11.12<br>15.81	14.01
**AUC**	RUN1: -10.78<br>RUN2: -12.11<br>RUN3: -16.60<br>RUN4: -15.76	10.64<br>9.31<br>4.82<br>5.66	7.6
**AUU**	RUN1: -3.63<br>RUN2: -6.93<br>RUN3: -9.40<br>RUN4: -5.3	17.8<br>14.49<br>12.02<br>16.12	15.1
**AUA**	RUN1: -4.40<br>RUN2: -7.94<br>RUN3: -3.17	17.02<br>13.48<br>18.25	17.37

	RUN4: -0.62	20.8	
**ACA**	RUN1: -8.77<br>RUN2: -6.85<br>RUN3: -6.10<br>RUN4: -7.47	12.65<br>14.57<br>15.32<br>13.95	14.12
**AGU**	RUN1: -5.07<br>RUN2: -4.56<br>RUN3: -4.92<br>RUN4: -5.82	16.35<br>16.86<br>16.5<br>15.6	16.32
**GUA**	RUN1: -5.7<br>RUN2: -2.82<br>RUN3: -2.06<br>RUN4: -4.09	15.72<br>18.36<br>18.6<br>17.33	17.50
**GCG**	RUN1: -3.03<br>RUN2: -4.80<br>RUN3: -3.92<br>RUN4: -2.26	18.39<br>16.62<br>17.5<br>19.16	17.9
**GCA**	RUN1: -0.6<br>RUN2: -1.82<br>RUN3: -2.01<br>RUN4: -3.94	20.82<br>19.6<br>19.41<br>17.48	19.32
**UAU**	RUN1: -2.22<br>RUN2: -0.9<br>RUN3: -2.31<br>RUN4: -1.54	19.2<br>20.52<br>19.11<br>19.88	19.67

The relative binding energies with respect to AUG codon were obtained $\left[\Delta\Delta E\right| \quad \left|Bind = \Delta E_{Bind}^{Mut} - \Delta E_{Bind}^{AUG}\right]$. ΔE_{Bind}^{AUG} is the average binding energy between codon AUG with anticodon UAC calculated using MMPBSA, over the four independent runs of AUG codon and the value is -21.42 kcal/mol. For each mutated codon, for each run relative binding energy is calculated with respect to AUG codon.

Supplementary Table 2: Binding energy values (kcal/mol) for different codons:anticodons in presence/absence of eIFs

CODON	No eIF1			No (eIF1+eIF1A)			No eIF2α			No eIF2β		
	Runs:	Rel:	Avg:	Runs:	Rel:	Avg:	Runs:	Rel:	Avg:	Runs:	Rel:	Avg:
AUG	-20.14<br>-23.53<br>-21.5<br>-21.42		-21.64	-19.92<br>-20.40<br>-19.02<br>-21.03		-20.09	-10.87<br>-17.69<br>-11.61<br>-10.5		-12.66	-16.6<br>-17.4<br>-18.2<br>-17.35		-17.38
GUG	-22.34<br>-18.11<br>-18.29<br>-19.9	-0.7<br>3.53<br>3.35<br>1.74	1.99	-25.30<br>-23.53<br>-24.22<br>-25.61	-5.21<br>-3.44<br>-4.13<br>-5.52	-4.57	-11.75<br>-6.60<br>-12.5<br>-14.5	0.91<br>6.06<br>0.16<br>-1.84	1.32	-14.33<br>-12.59<br>-15.03<br>-15.4	3.05<br>4.79<br>2.35<br>1.98	3.04
AUA	-13.24<br>-13.68<br>-13.82<br>-14.10	8.4<br>7.96<br>7.82<br>7.54	7.93	-14.85<br>-14.08<br>-13.28<br>-14.51	5.24<br>6.01<br>6.81<br>5.58	5.91	-7.85<br>-15.1<br>-8.9<br>-7.2	4.81<br>-2.44<br>3.76<br>5.46	2.89	-5.62<br>-6.18<br>-7.09<br>-6.16	11.76<br>11.2<br>10.29<br>11.23	11.12

Relative binding energy profile of codon-anticodon interactions from GUG and AUA with respect to AUG simulation runs in the presence and absence of different eIFs. For GUG and AUA, for each case, relative binding energy is calculated with respect to AUG codon for the similar system.
