# Supplementary material for: Selection of start codon during mRNA scanning in eukaryotic translation initiation
Source: Commun Biol. 2022 Jun 15;5:587. doi: 10.1038/s42003-022-03534-2 (PMC9200866; doi:10.1038/s42003-022-03534-2)
Supplement: Supplementary file 3 — Description of Additional Supplementary Files [file 42003_2022_3534_MOESM3_ESM.pdf]

Description of Additional Supplementary Files

File Name: Supplementary Data 1
Description: This file contains the binding energy values (kcal/mol) for different codons:anticodons for Figure 2. The relative binding energies with respect to AUG codon were obtained. The average binding energy between codon AUG with anticodon UAC calculated using MMPBSA, over the four independent runs of AUG codon and the value is -21.42 kcal/mol. For each mutated codon, for each run relative binding energy is calculated with respect to AUG codon.

File Name: Supplementary Data 2
Description: This file contains binding energy values (kcal/mol) for different codons:anticodons in presence/absence of eIFs for Figure 3. Relative binding energy profile of codon-anticodon interactions from GUG and AUA with respect to AUG simulation runs in the presence and absence of different eIFs. For GUG and AUA, for each case, relative binding energy is calculated with respect to AUG codon for the similar system.

File Name: Supplementary Data 3
Description: This file contains Root mean square deviation values of tRNAi backbone from independent simulation runs of AUG (blue), AUA (green) and GUG (red), respectively used for Figure 5a.

File Name: Supplementary Data 4
Description: This file contains Root mean square fluctuations of atoms in nucleotides of the anticodon of tRNAi in the case of AUG (blue), GUG (red) and AUA (green) MD simulation run used for Figure 5b.

File Name: Supplementary Data 5
Description: This file contains root mean square fluctuation of eIF2α atoms used for Figure 6d.
